# Bio-inspired apparatus to produce luminescent cavitation in a rigid walled chamber

**DOI:** 10.1371/journal.pone.0293839

**Published:** 2023-12-14

**Authors:** Samuel Cruz, Francisco A. Godínez, Luis Enrique Martínez-Alvarado, Rubén Ramos-Garcia

**Affiliations:** 1 Departamento de Óptica, Instituto Nacional de Astrofísica, Óptica y Electrónica, Puebla, México; 2 Instituto de Ingeniería, Unidad de Investigación y Tecnología Aplicadas, Universidad Nacional Autónoma de México, Apodaca, Nuevo León, México; 3 Facultad de Ingeniería, Universidad Nacional Autónoma de México, Ciudad de México, México; The British University in Egypt, EGYPT

## Abstract

A mechanical device inspired by the rapid rotational motion of the pistol shrimp plunger has been developed to experimentally study the contraction/expansion dynamics of a gas bubble inside a confined liquid volume and in the vicinity of solid surfaces. The apparatus consists of a limb with a V-shaped end, which fits into a socket forming a cylindrical compression chamber. Air bubbles of different sizes and in different positions inside the chamber were seeded to study their shape evolution in liquids when subjected to pressure pulses induced by the limb closure. By changing the standoff and curvature parameters, as well as the closing power of the limb it was possible to control the dynamical behavior of the cavity. Four stages describing the dynamic behavior of the bubble were found: 1) A slight expansion-contraction stage accompanied by very weak volumetric oscillations. 2) First compression stage. The formation of gas and liquid micro-jets is observed when the vertical symmetry axis of the bubble is initially located outside of the chamber symmetry axis, on the other hand, when there is a coincidence between these axes, the bubble only contracts exhibiting non-spherical shapes, alternating between oblate and prolate spheroidal structures. 3) An expansion stage where the cavity reaches the walls of the chamber exhibiting irregular shapes on its surface. 4) Second compression stage. This process begins when the limb rebounds and stops sealing the chamber allowing a jet of liquid to enter from the fluid medium outside, inducing a very violent collapse accompanied by the emission of light. The proposed technique represents a novel alternative to study the dynamic evolution of bubbles near and on solid boundaries of various geometries. Other attractive features of the apparatus are its low manufacturing cost, simple design and compact size which makes it easily portable.

## Introduction

Over decades several devices and procedures have been developed to produce and study cavitation and associated phenomena such as the generation of chemical reactions, shock waves and luminescence. Cavitation events are interesting and of great importance, because they occur in countless applications and industrial processes; sometimes representing an advantage as in the cleaning of water [1]; but in other situations representing an undesirable phenomenon because of its harmful effects as in the erosion of propellers in ships [2]. The first example of an apparatus for producing cavitation bubbles of several tens of millimeters is based on the tube arrest method. The idea is to accelerate a liquid-filled tube and then suddenly stop it, thus generating an abrupt deceleration and the appearance of bubbles at the bottom of the pipe [3]. Another interesting system to study the collapse of gas bubbles is the so-called U-tube device. In this case, a certain amount of fluid, acting as a liquid piston, is poured into the tube; one of the arms of the pipe ends with a conical chamber into which a certain amount of gas is injected, while the other arm is pressurized with a step excitation causing the liquid piston to accelerate and compress the bubble in the conical pocket [4]. The Venturi tube is another device used to produce bubbles for various applications [5]. The hydrodynamic cavitation generated with Venturi profiles is due to variations in fluid velocity/pressure induced by changes in the geometry of the channel. In other devices acoustic energy is harnessed to produce cavitation, a common arrangement employs a resonator vessel driven by piezoelectric transducers to create an acoustic field by which bubbles can be trapped and made to oscillate volumetrically. The collapse of the cavities becomes so violent that the generation of shock waves and photon emission is possible [6, 7]. In addition to acoustic and hydrodynamic energies, it is possible to use different sources to generate cavities. A laser depositing its energy at a point within a fluid produces rapid evaporation and thus the growth and collapse of bubbles [8–10].

Other possibilities are the use of micro explosives [11] and electrical discharges [12, 13] in liquids to produce sudden evaporation and consequently the genesis of bubbles. One aspect to note is that all of the above-mentioned devices and procedures allow, under certain circumstances, the generation of luminescent cavities. According to the literature review presented above, there is a lack of development of techniques and methodologies (alternative to the conventional ones) to produce cavitation and study its effects. In particular, there is a need for devices of low-cost and simple design that enable low-cost research focused not only on basic science but also on education. In this investigation, a different alternative to induce collapse and luminescence of gas bubbles is explored. The main objective focuses on developing and experimentally testing a bio-inspired mechanical device to study the contraction/expansion dynamics of a gas bubble inside a bounded liquid volume. The apparatus is equipped with a rotating limb and a cylindrical rigid walled chamber, this basic design mimics the sudden rotational motion of the pistol shrimp’s plunger and the appendages of the mantis shrimp. It is well known how these animals take advantage of this evolutionary adaptation to produce cavitation for defense and hunting [14, 15]. The proposed design also integrates ideas from the operation of the U-tube device. But instead of using a liquid piston, one of the ends of the rotating limb (in the form of a V-tip) is used as a solid plunger that compresses fluid and seals the cylindrical compression chamber where a bubble is seeded. In addition to the primary goal stated above, four questions were formulated to guide the research: 1) How does the spherical shape of the bubble initially seeded in the compression chamber evolve when subjected to a pressure pulse induced by the sudden closure of the limb? 2) What is the effect of the initial position of the bubble inside the chamber? 3) What effect does the limb closure speed have on the characteristics of the light pulses coming from bubble collapses? 4) What effect does the initial size of the bubble have on the characteristics of the light flashes emitted during its collapse?

The answers to each of these questions are discussed in detail in later sections. In general terms, however, this study shows how the proposed device is useful to produce the collapse of gas cavities, whose initial size and position inside the chamber can be controlled. The collapse velocities along with the shape evolution can be modified by changing the position and size of the bubbles as well as the angular velocity of the limb. It was also shown that the collapse of the bubbles is accompanied by photon emission and that the intensity and width of the light pulses can be modified by using a mixture of water and chlorine dioxide, which was particularly useful for enhancing luminescence.

## Device design

**Fig 1:**
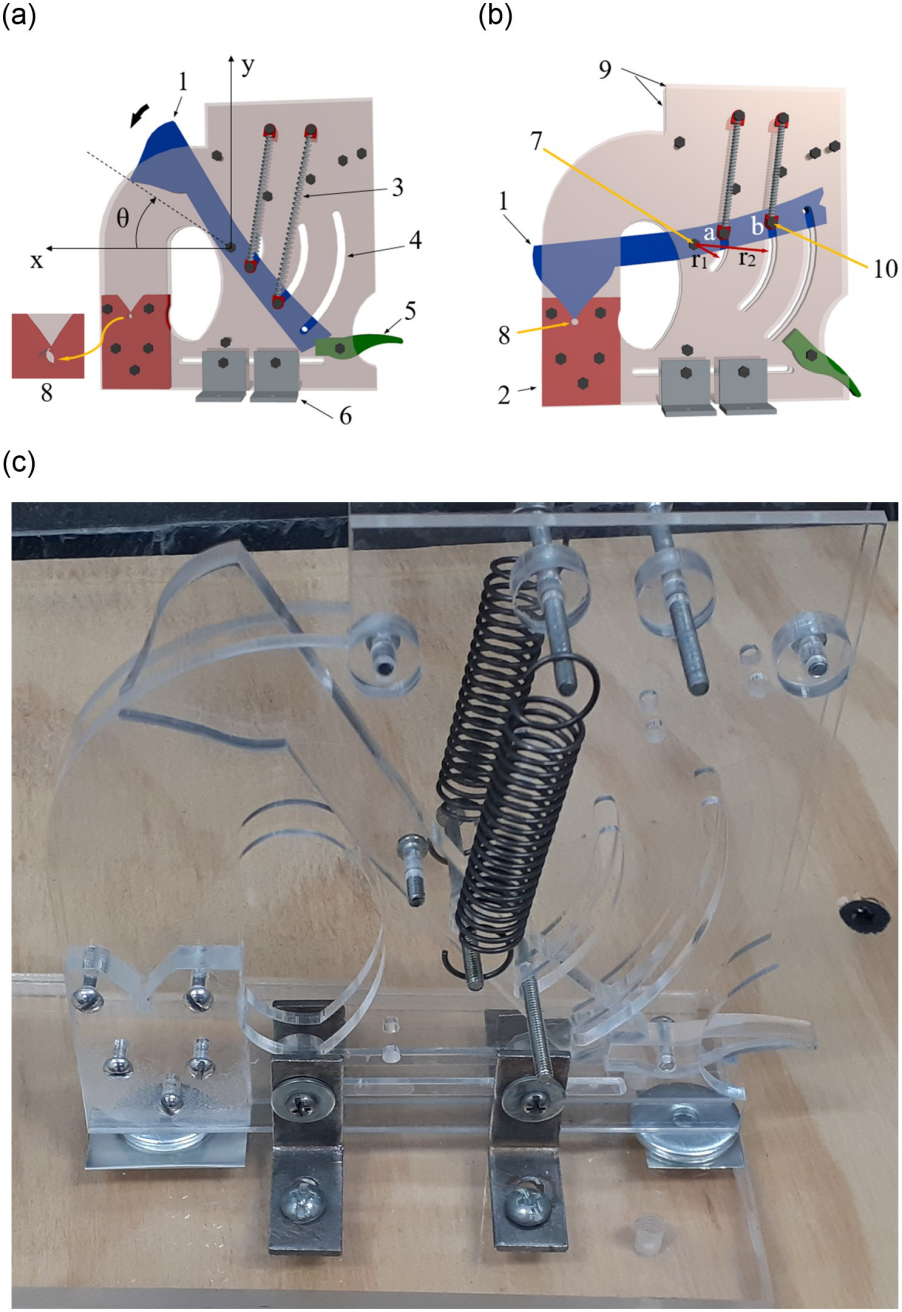
3D CAD model of the apparatus. (a) in open position with springs stretched and the limb locked with the trigger and (b) in closed position just when the limb has reached the end position of its travel. The device consists of: (1) a limb, (2) a lower fixed part, (3) a plurality of tension coil springs, (4) a set of curved grooves, (5) a mechanical trigger, (6) a set of fastening brackets, (7) a pivot, (8) a cylindrical compression chamber, (9) a pair of parallel plates, (10) a set of screws to secure the springs to the limb. Note that the positive direction of the angular displacement, *θ*(*t*), was taken in a clockwise orientation. (c) Picture of the device built with acrylic plate (polymethyl methacrylate, PMMA) using the laser cutting manufacturing technique.

Fig 1 shows two views of the 3D CAD model of the proposed apparatus. It should be noted that the limb (1) has a rectangular slot at its lower end to facilitate the locking of this element with the trigger (5), as can be seen in Fig 1a. The other end of the limb has a V-shape, which fits perfectly with the V-shape of the fixed lower part (2). However, the tip of the V-shape of the fixed element (2) ends in a circular contour, which together with the side plates (9) make up the cylindrical compression chamber (8). The device works when it is immersed in a liquid with the limb in the maximum opening position. Then a bubble of gas is seeded inside the compression chamber. When the trigger is activated, the limb is released and it undergoes a sudden angular acceleration around the pivot (7), due to the contraction of the springs (3), describing a circular trajectory in the direction indicated by the curved arrow in Fig 1a. As the limb closes, a pressure increase is generated in the fluid inducing the compression of the bubble. In the final stages of the closure process, the V-shape end of the limb fits that of the fixed lower part, partially sealing the cylindrical chamber, see Fig 1b. It is worth mentioning that the curved grooves (4) are used to guide the clamping screws (10) and, in addition, the ends of these grooves serve as mechanical stops, slowing down the movement of the screws (10) and of the limb.

It is pertinent to highlight some advantages that our device has over other systems: 1) It is built with low-cost materials and manufacturing techniques, transparent acrylic and laser cutting, respectively; 2) it does not require additional equipment or facilities as is the case with other devices and techniques; 3) the design of both the limb and compression chamber can be easily modified; 4) it is built on a cm scale, so it takes little space, although larger versions can of course be used.

### Kinematic behavior of the limb

**Fig 2:**
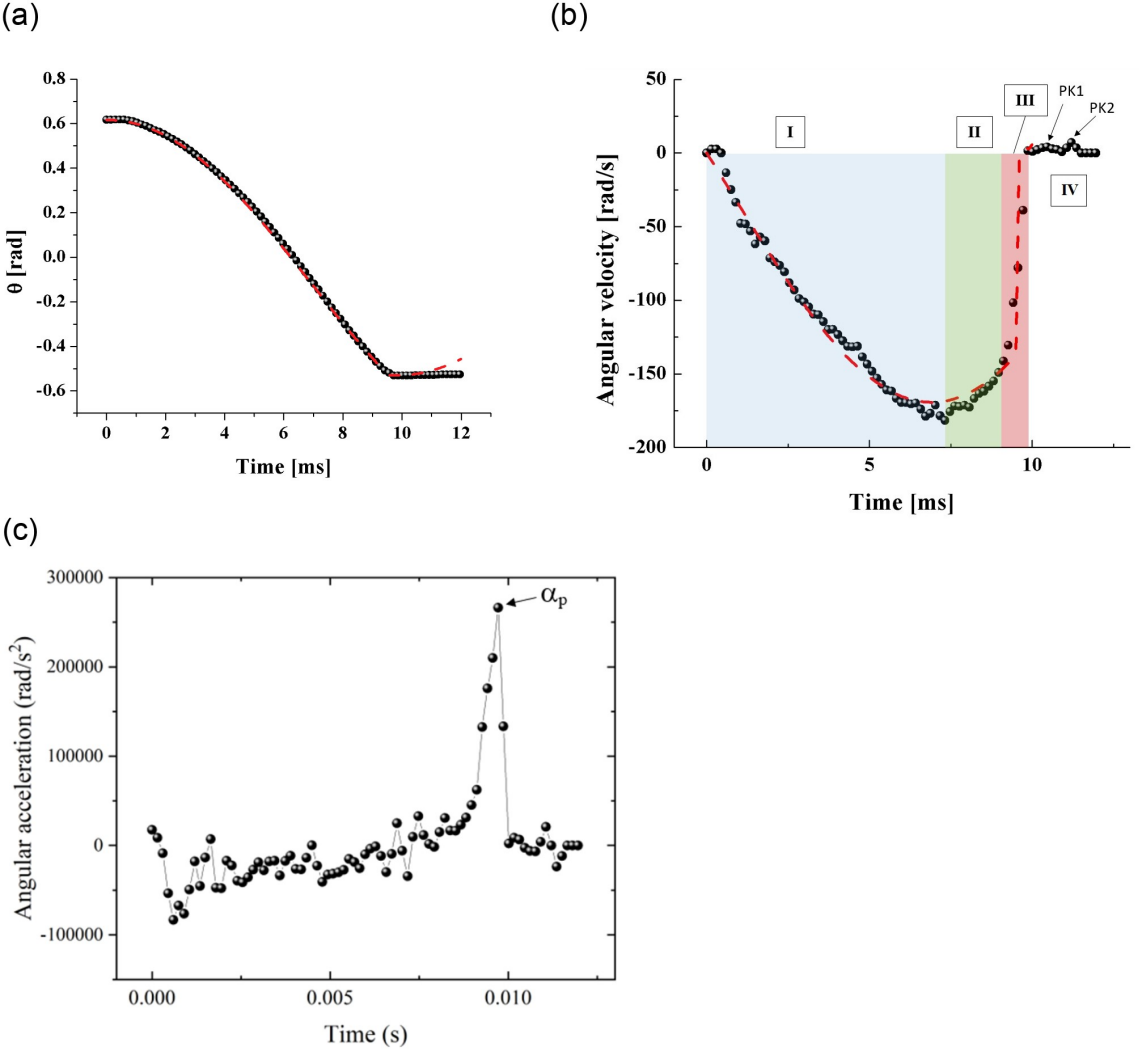
(a) Angular displacement, *θ*(*t*), developed by the arm from the initial position when it is released until it reaches the final closing position, (b) angular velocity of the limb (in this figure the peaks of the first, PK1, and second, PK2, bounces are shown). (c) Angular acceleration vs. time graph developed by the limb. Four springs were used to obtain these experimental curves.

Fig 2 shows the angular displacement developed by the limb from the moment it is released until it reaches the final closing position. The motion of the limb was generated by the contraction of four tension springs. Experimental data (symbols) were obtained by analyzing high-speed videos taken at 73,000 fps with an exposure time of 10 *μ*s. Both the displacement and the angular velocity of the limb can be simulated using a model in terms of a balance of torques around the pivot (7). Thus, according to Newton’s second law it is easy to find the differential equation of the limb dynamics [16, 17]:

$$\begin{array}{c} \begin{array}{c} I_{zz}\frac{d^{2}\theta \left(t\right)}{dt^{2}}=-K\theta \left(t\right)+C_{D}{\left(\frac{d\theta (t)}{dt}\right)}^{2}+\beta \frac{d\theta (t)}{dt} \end{array} \end{array}$$ (1)

subject to the initial conditions

$$\begin{array}{c} \begin{array}{c} \theta \left(t=0\right)=\theta_{0},\;{\;\frac{d\theta (t)}{dt}|}_{t=0}=0 \end{array} \end{array}$$ (2)

where *θ*(*t*) is the angular displacement from rest position, *K* represents a linear torsional stiffness, *C*_*D*_ stands for a drag coefficient in which the fluid properties and the geometry of the limb are included, and *β* is a linear viscous damping parameter. *I*_*zz*_ is the moment of inertia about the rotation axis (estimated with Solid Edge software). The dashed lines in Fig 2a and 2b are the best fits of the model represented by Eqs 1 and 2 to the experimental data. The fitting process was performed by applying the least squares technique in Wolfram Mathematica 10.0. The computations were carried out with the following data: *I*_*zz*_ = 44 × 10^−6^kg⋅m^2^, *θ*_0_ = 0.617 rad, *θ*_*f*_ = −0.527 rad. In this way we determined the values of *K* = 2.5 N⋅m/rad, *C*_*D*_ = 3 × 10^−6^ kg⋅m^2^ and *β* = 2.2 × 10^−3^kg⋅m^2^/s.

It is interesting to note how the angular velocity developed by the limb shows four clearly identifiable phases. An acceleration stage (I), indicated with a blue region, in which the limb reaches a maximum velocity (this stage lasts about 8 *ms*), then a gradual deceleration stage (II) that lasts about 1 *ms* is observed (green region); next, a sudden deceleration (III) characterized by a sharp velocity drop and consequently a short duration ∼0.5 *ms* is noticed (red region); finally, a region of tiny bounces (IV) at the end of the process is identifiable. It is noteworthy that a similar behavior has been experimentally observed in mechanical devices with pivoting elements [16–19]. It is also of value to compare the limb peak velocity developed by our device and that by the biological systems. The pistol shrimp is capable of developing velocities of ∼ 10 *m*/*s* [20] and the mantis shrimp can reach values of up to 20 *m*/*s* [21]. Our device using an arrangement of four tension springs is able to develop velocities of about 9.5 *m*/*s*, very close to those of the real pistol shrimp. The velocity of the artificial system can, in principle, be increased by employing stiffer springs and/or a larger number of these elastic elements, however, the drag forces that will have to be counteracted cannot be overlooked.

### Design calculations and simulations

During the operation of the mechanism, the limb rotates at high speed due to the contraction of the springs until it completes its travel when it is abruptly braked by impact forces (or torques) between the clamping screws (10) and the internal rounded zone at each end of the curved grooves (4) of the parallel plates (9), see Fig 1a and 1b. A structural analysis was carried out to determine the stresses to which the elements of the mechanism are subjected during operation. The maximum braking torque *T*_*m*_ (caused by the collision forces and their respective lever arms *r*_1_ and *r*_2_, as shown in Fig 1b) due to the deceleration produced by the impact forces is estimated as:

$$\begin{array}{c} \begin{array}{c} T_{m}=I_{zz}\alpha_{p}=\left(44\times 10^{-6}Kg\cdot m^{2}\right)\left(266,000\frac{rad}{s^{2}}\right)=11.704N\cdot m, \end{array} \end{array}$$ (3)

where *I*_*zz*_ is the moment of inertia, while the value of the angular deceleration *α*_*p*_ was the highest value (the peak) calculated from the second derivative of the angular displacement-time curve developed by the limb, see Fig 2c.

Assuming that *T*_*m*_ is divided equally, i.e., a torque with a lever arm r1 corresponding to screw (a) and a torque with a lever arm r2 corresponding to screw (b), one can calculate:

$$\begin{array}{c} \begin{array}{c} T_{m}=T_{1}+T_{2}, \end{array} \end{array}$$ (4)

$$\begin{array}{c} \begin{array}{c} T_{1}=T_{2}=5.852N\cdot m. \end{array} \end{array}$$ (5)

From the definition of torque, *T*_*i*_ = *F*_*t*,*i*_*r*_*i*_; *i* = 1, 2, the tangential force acting on each screw can be calculated as:

$$\begin{array}{c} \begin{array}{c} F_{t,1}=\frac{T_{1}}{r_{1}}=454.7N, \end{array} \end{array}$$ (6)

$$\begin{array}{c} \begin{array}{c} F_{t,2}=\frac{T_{2}}{r_{2}}=174.7N. \end{array} \end{array}$$ (7)

**Fig 3:**
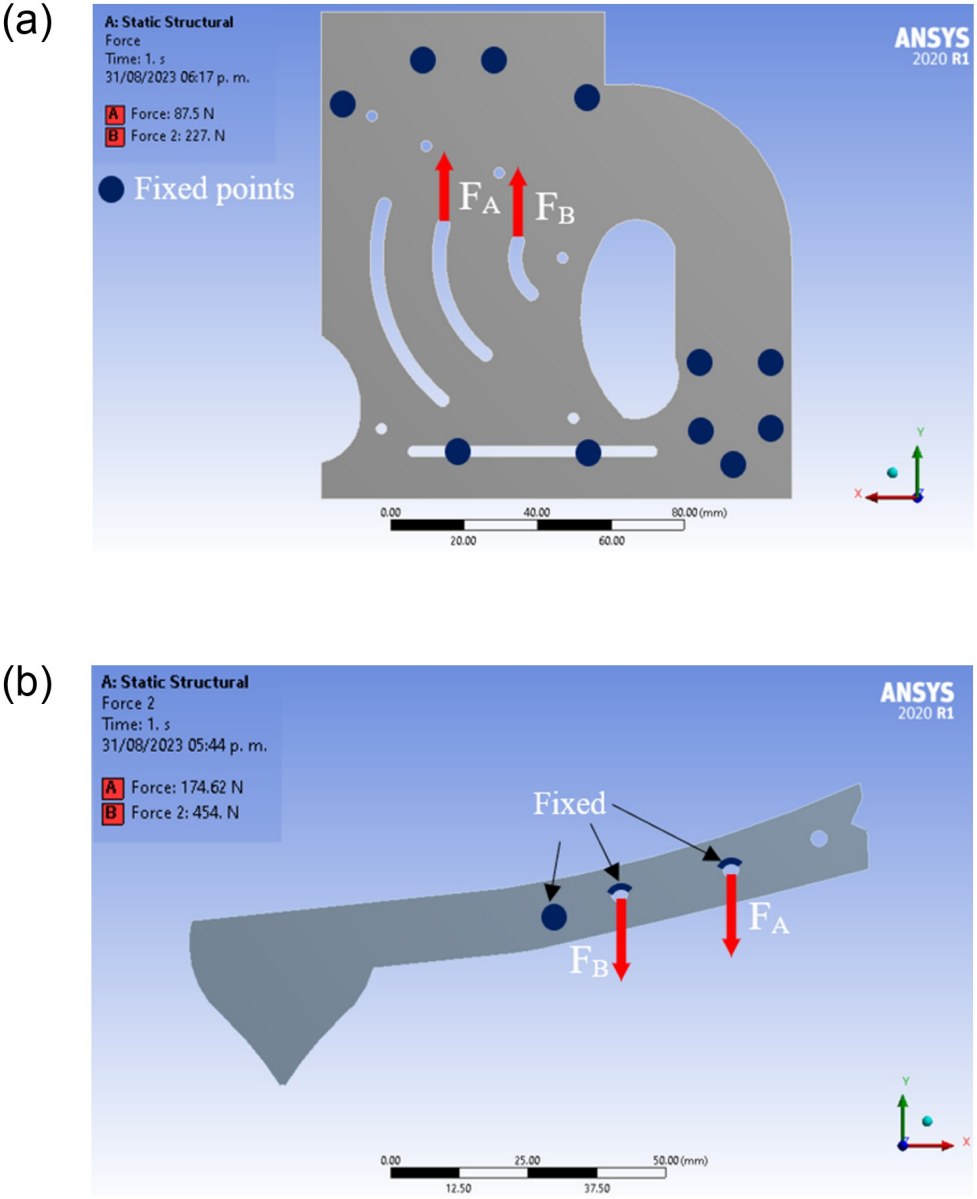
Forces and fixed points considered for the stress-strain simulations on the (a) parallel plates and (b) on the limb.

Based on these forces, the structural static analysis was performed in the ANSYS 2021 Workbench module. The elements experiencing the highest impact forces are the parallel plates (side walls) of the mechanism and the limb. The points at which these forces are applied and their respective directions, as well as the boundary conditions, are shown in the diagrams in Fig 3a and 3b.

The parallel plates and the limb were meshed with quadratic elements with element sizes of 1 mm and 0.5 mm in the areas where the impact occurs. Since there are two plates supporting the impact force of the limb, the tangential forces calculated with Eqs 6 and 7 were divided by two:

$$\begin{array}{c} \begin{array}{c} \frac{F_{t,1}}{2}=F_{B}=227.4N, \end{array} \end{array}$$ (8)

$$\begin{array}{c} \begin{array}{c} \frac{F_{t,2}}{2}=F_{A}=87.1N. \end{array} \end{array}$$ (9)

Fig 3a shows the forces A and B supported by the plates. The motion of the plates was also restricted at the points indicated in blue, which coincide with the points that were fixed in the experiments. The direction of the forces for the limb is opposite to the one set in the plates due to Newton’s third law of action/reaction, see Fig 3b. For this case, the forces calculated with Eqs 8 and 9 act entirely on the limb, thus *F*_*t*,1_ = 2*F*_*B*_ and *F*_*t*,2_ = 2*F*_*A*_. Also this figure shows the regions where the limb motion was restricted.

**Fig 4:**
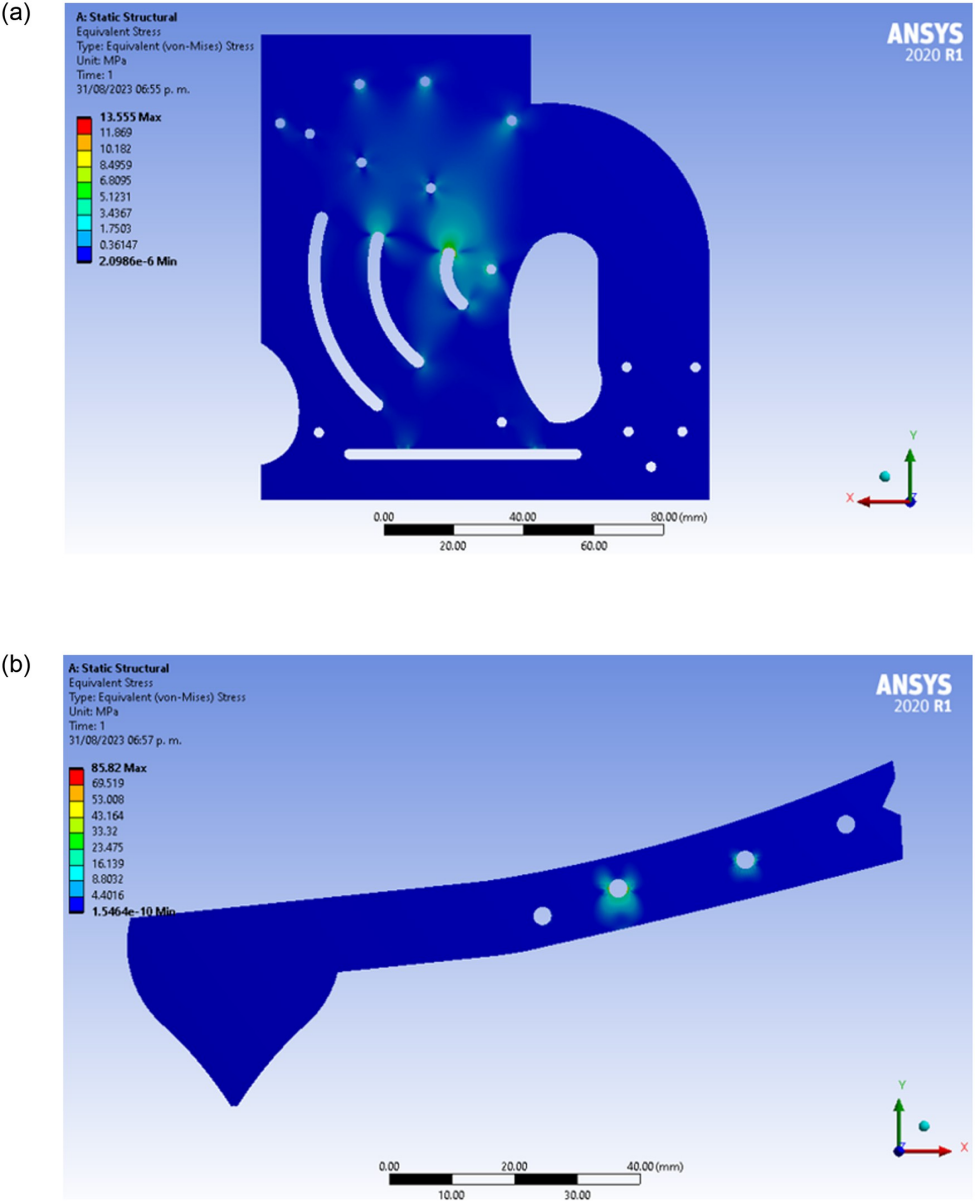
Stress distribution from numerical simulations for (a) the plates and (b) for the limb.

The results of the stress simulations are shown in Fig 4a and 4b. Using the von Mises stress failure criterion, it is observed that, both in the walls and at the limb, the highest stresses are concentrated in the regions close to the impact sites.

When comparing the highest von Mises stresses calculated against the elastic limit of the material (86 MPa [22]), it is observed that both in the plates and in the limb, the stresses generated fall within the elastic limit, since *σ*_*von*−*Mises*_ < *σ*_*Yield*_. However, it is important to mention that the safety factors for the wall and for the limb are different. Defining the safety factor, SF, as:

$$\begin{array}{c} \begin{array}{c} SF=\frac{\sigma_{Yield}}{\sigma_{von-Mises}}, \end{array} \end{array}$$ (10)

The design of each plate (wall) has a SF of:

$$\begin{array}{c} \begin{array}{c} SF=\frac{86M\;Pa}{13.55M\;Pa}=6.37, \end{array} \end{array}$$ (11)

while the limb design has a SF of:

$$\begin{array}{c} \begin{array}{c} SF=\frac{86M\;Pa}{85.82M\;Pa}=1.002. \end{array} \end{array}$$ (12)

A low SF (as estimated for the limb) indicates that the material is working right at the elastic limit, but at no time does it indicate failure. For failure to occur, the stresses reached in the material would have to exceed its ultimate stress, which in this case is 100 MPa.

It is important to mention that the design of the apparatus as presented in this research represents a low-cost laboratory prototype. The acrylic (polymethyl methacrylate, PMMA) used for its construction presents a good chemical resistance to water and to the mixture of water and chlorine dioxide used in the experiments. In addition, the mechanical strength of acrylic allows dozens of experiments to be carried out without structural damage. However, it must be recognized that the proposed design has limitations: 1) it should not be used with corrosive chemicals that are particularly aggressive to acrylic such as agents containing benzene, ethanol and other alcohols, organic materials or thinners, 2) it is not suitable for working with abrasive liquids that may modify the roughness and thus the transparency of acrylic (which would hinder the visualization of bubble dynamics), 3) it should not be used with liquids at high temperatures (at most it should operate at 60 °C), 4) the elements of the device will generally not withstand high impact forces. Thus the factors affecting the performance of the device are primarily the type of liquid used and its temperature, as well as the stress levels to which the acrylic is subjected during the impact forces in the final stages of the limb closure. Now, if the design requirements were to focus on developing an apparatus with a high level of chemical/mechanical resistance and high durability (fatigue resistance), it would be appropriate to use some type of stainless steel for its construction. Of course, this would imply much higher raw material and manufacturing costs.

## Experimental setup

**Fig 5:**
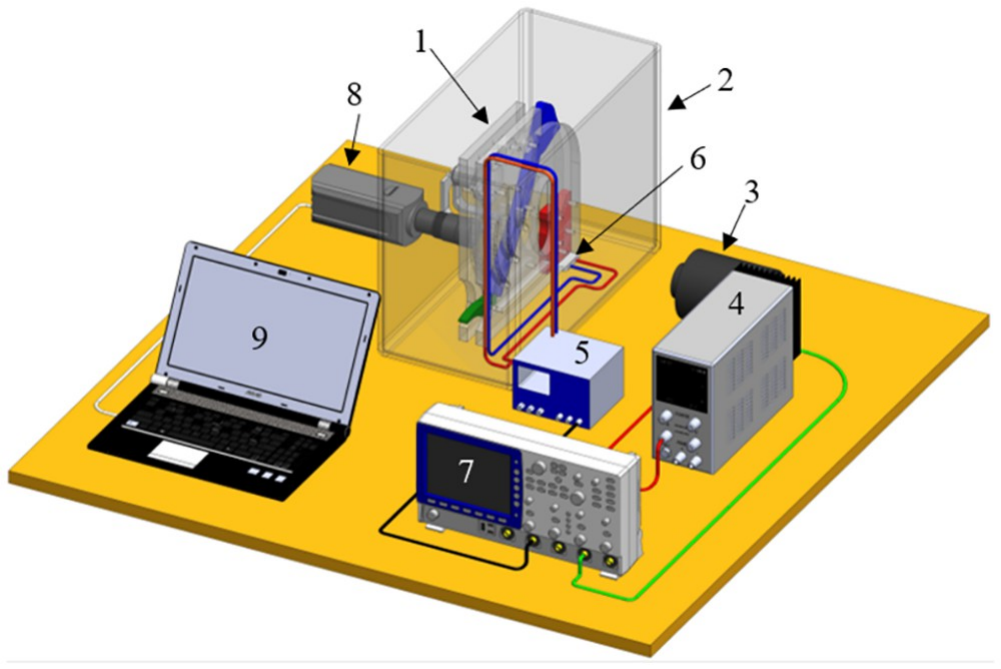
Experimental setup. (1) Bio-inspired device, (2) container, (3) photo-multiplier, (4) high voltage source for the photo-multiplier, (5) conditioner/amplifier for PVDF sensor signal, (6) PVDF sensor, (7) oscilloscope, (8) high-speed video camera, (9) PC to control the camera.

The apparatus was set inside a rectangular container (44 × 44 × 22) *cm*^3^. A PVDF film sensor (DT1–028K W/TH), placed under the base of the device, was used to measure mechanical vibrations and acoustic disturbances generated by the bubble dynamics. One Phantom V7.3 camera (equipped with focusing optics SIGMA APO MACRO DG, 180mm 1:3.5) along with two 50 Watt halogen lamps were used to capture cavitation dynamics. In addition, an ET Enterprises photo-multiplier (model 9354KB) was used to detect light emissions. All signals from the sensors were acquired with an oscilloscope (Tektronix TD55104B). The experimental setup used for the tests is depicted in Fig 5.

### Materials and methods

All experiments were performed at room conditions (77 kPa pressure and 23 °C temperature). Distilled water (*P*_*v*_ = 3 kPa, *ρ* = 1036 kg/m^3^, *ν* = 1 cSt, 0.2 *μ*/cm and 0.1 ppm) and a mixture of water with chlorine dioxide (*P*_*v*_ = 104 kPa, *ρ* ≈ 1000 kg/m^3^ and *ν* ≈ 1 cSt) were used as working fluids. The aqueous solution was prepared with a chlorine dioxide concentration of 20 milligrams per liter. The mixing was carried out with a magnetic stirrer for 5 minutes to homogenize. It is noteworthy that the chlorine dioxide (*ClO*_2_) is used into food processing, to transfer oxygen to a variety of substrates, water treatments, among others [23, 24].

## Results and discussion

### Photographic sequence of limb motion, light and vibration signals

**Fig 6:**
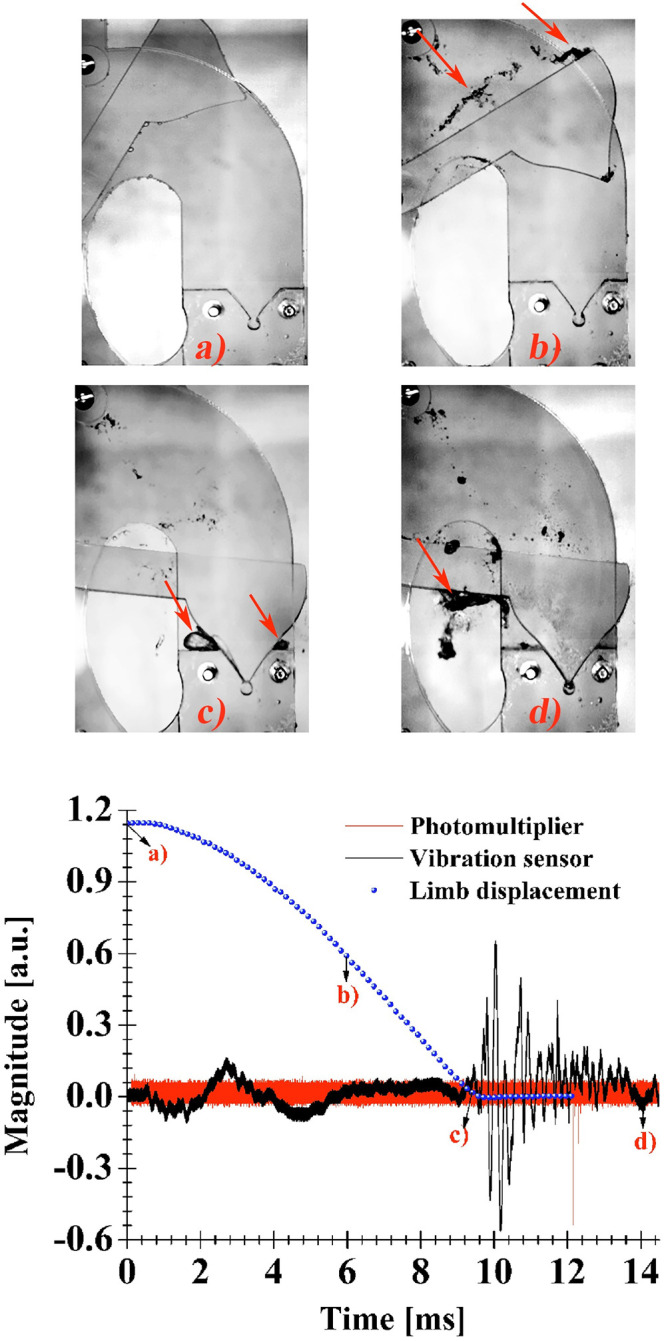
Top: High-speed photographic sequence of the Limb’s motion. Bottom: Light and vibration signals from the photomultiplier and the PVDF sensor, respectively.

Fig 6 shows the angular displacement of the limb (using four springs) together with the light and vibration signals from the sensors. When the limb is released, its sudden rotation starts (a), after a few moments, the upper part of the limb generates cavitating structures in its interaction with the fluid (b). These structures are dragged along with the limb leaving a wake (indicated by arrows) of bubbles that lasts for about 4 ms. Subsequently, the V-tip reaches the final position partially sealing the compression chamber (c), around this moment, on one hand a pair of vapor lobes (indicated by arrows) are generated, on each side of the lateral faces of the V-shaped limb end; on the other hand, the seeded bubble in the cylindrical chamber collapses emitting photons and pressure waves. Note, that the oscillations of greater amplitude observed in the vibration sensor signal, after the instant (c), are due to the impact between the ends of the curved grooves (4) and the screws (10) attached to the middle and lower parts of the limb (see Fig 1a and 1b), as well as the collapse of the seeded bubble. The instant (d) marks the end of the process, only the remnant, in the form of bubble clouds, of the lobes collapses is visible (indicated with an arrow). Our investigation focuses mainly between instants (c) and (d), corresponding to stages III and IV in Fig 2b.

**Fig 7:**
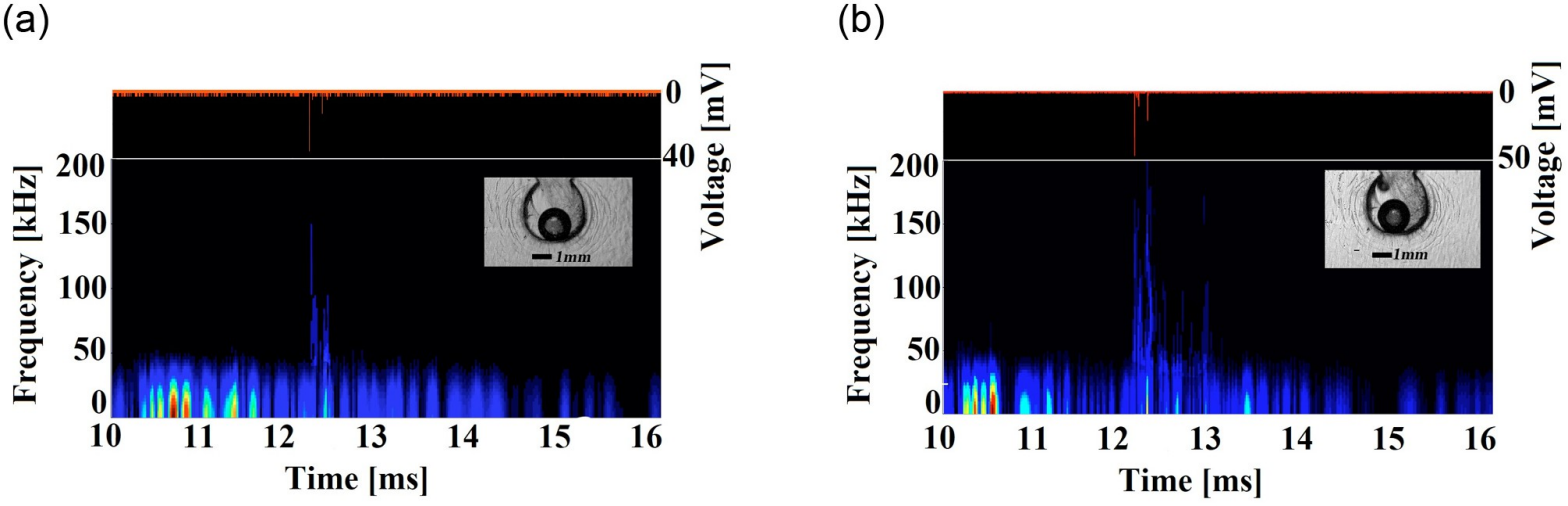
Light emissions and spectral analysis from the vibration sensor for a bubble in (a) water and in (b) water with chlorine dioxide. Both bubbles are characterized by same standoff and curvature parameters *γ* = 1, *ξ* = 2. In both cases, vibrations with frequencies as high as 200 kHz, as well as light emissions after the sudden limb stop are generated. In addition, the mixture of water and chlorine dioxide clearly enhances the magnitude of light emissions and increases the number of pulses.

The spectral analysis of the vibration sensor and the light emission signals generated at stage IV are presented in Fig 7, for both (a) water and (b) water with chlorine dioxide. Note that there is a bubble seeded with a similar diameter in each case, so the standoff and curvature parameters (*γ* = 1, *ξ* = 2) are similar. The two cases have a similar spectral density since the limb dynamics exhibits practically the same behavior. The occurrence of vibrations at frequencies as high as 200 kHz is clear. These vibrations could be related to bubble collapses and coincide with the time of light emission for both cases. On the other hand, the intensity and number of light emissions differ in both cases; clearly, the addition of chloride dioxide enhances light intensity.

**Fig 8:**
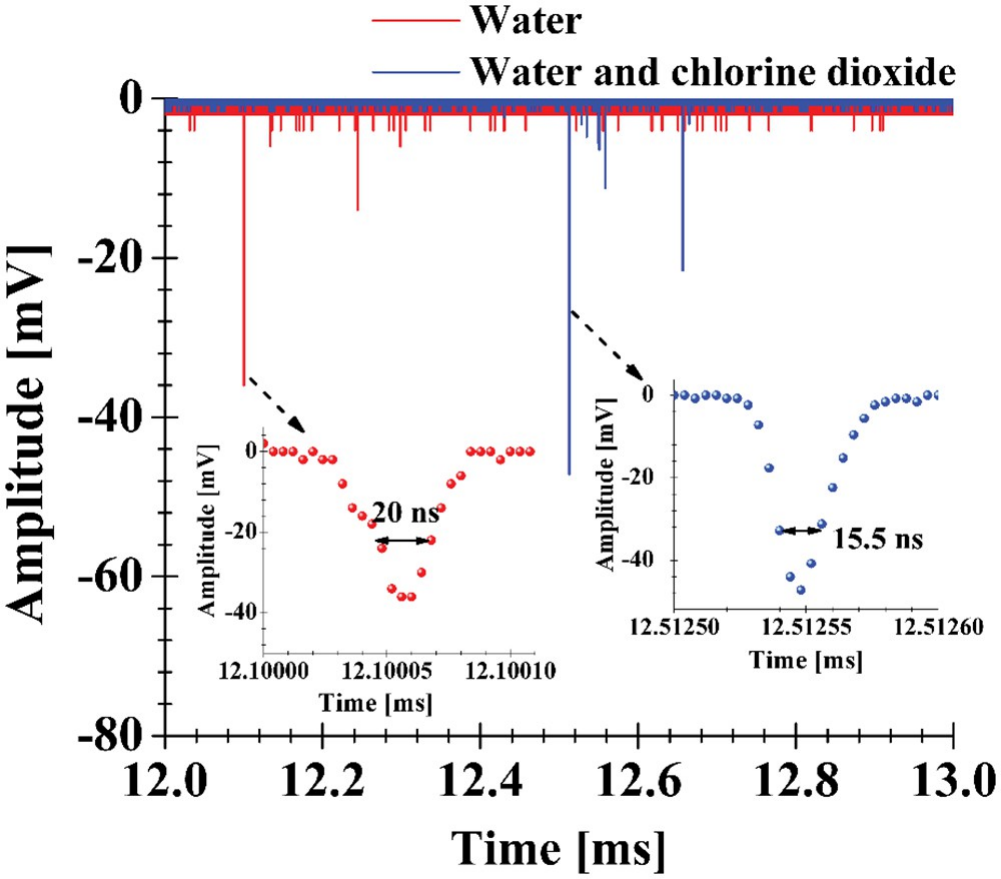
Enlarged view of the light emission pulses for water and for the mixture of water and chlorine dioxide. The FWHM is indicated in each pulse.

Fig 8 shows an enlargement of the light emission signals for both working fluids. Evident differences between the emission times, magnitudes and duration of the pulses are observed. In addition to boosting the intensity of the emissions, small concentrations of chlorine dioxide in water shorten the full width at half maximum of the pulse (FWHM), for water 20 ns and for the mixture 15 ns. We believe that the causes of these differences could be investigated/explained by spectroscopic measurements/analysis; however, this is beyond the scope of the present work. Similar FWHM for light emissions from collapses of laser-induced bubbles (10–20 ns) and from collapsing cavities generated with bio-inspired mechanisms are observed (8–24 ns) [19, 25]. Moreover, multiple peaks were observed, which could be associated with different hot spots in the inhomogeneous interior of the bubble and with its possible fragmentation into smaller collapsing cavities [25].

### Shape evolution of bubbles in water and in a mixture of water and chlorine dioxide

**Fig 9:**
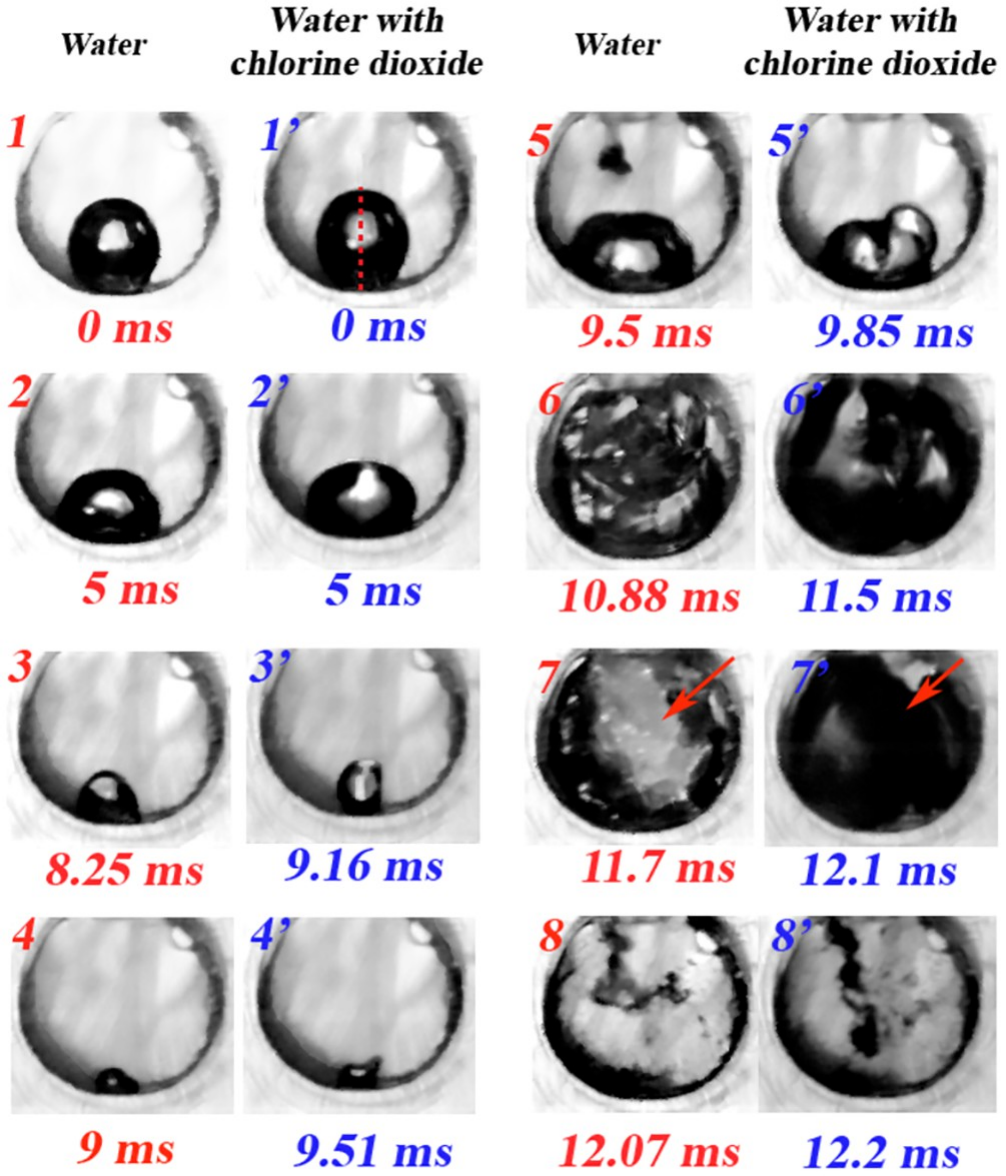
High-speed photographic sequences of bubble dynamics in water and in the water/chlorine dioxide mixture. The evolution of the bubble volume is similar in both cases (*γ* = 1, *ξ* = 2). The arrows indicate the region where the liquid jet enters from the outside when the limb recoils and stops sealing the chamber.

**Fig 10:**
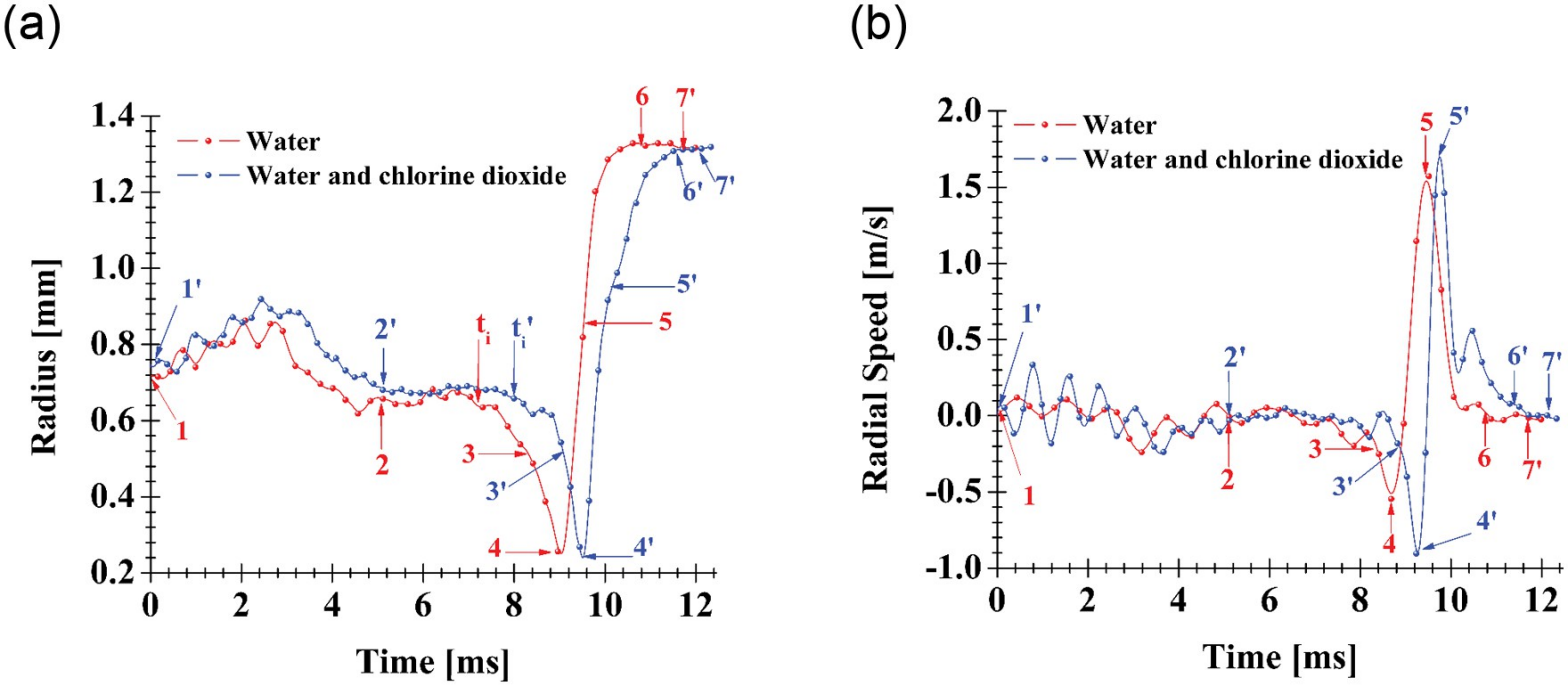
(a) Bubble size evolution in the vertical direction and (b) bubble size speed in the vertical direction as a function of time. Measurements were performed for bubbles (with diameter *ϕ* ≈ 1.5 mm, *γ* = 1, *ξ* = 3) seeded in water and in water with chlorine dioxide. The numbers indicated on the curves correspond to the instants in Fig 9.

Fig 9 displays photographic sequences of the shape evolution of two bubbles with similar initial conditions (diameter *ϕ* ≈ 1.5 mm, *γ* = 1, *ξ* = 2), one is immersed in water and the other in the mixture. When the limb is set in motion, both bubbles exhibit a series of surface oscillations and at the same time both gradually increase in size until they reach a maximum and then slowly contract (instants 1 to 2 for the bubble in water, 1’ to 2’ for the bubble in the mixture). It is important to note that at the beginning of the process the bubbles slightly resemble a prolate spheroid and end up with an oblate spheroid shape. At instants (*t*_*i*_, $t_{i}^{\prime }$), indicated in Fig 10a, a significant increase in the bubble contraction rate is observed, then two consecutive changes in the shape of the spheroids from oblate (2 and 2’) to prolate (3 and 3’) and vice versa are observed to finally reach the minimum volume (4 and 4’), this process is referred to as the first compression stage. After the bubbles have reached the minimum size, a rapid expansion is observed due to the low pressure caused by the initial recoil (see first peak in Fig 2b stage (IV)) of the limb (see 5 and 5’). This causes an unstable behavior on the interface of the bubbles and thus they are unable to maintain their spherical shape [26]. The maximum size that the bubbles reach is apparently (we could only visualize the side face of the chamber) the total volume of the compression chamber (see 6 and 6’). This condition remains by almost 2 ms until the limb recoils a second time, see second peak in Fig 2b stage (IV), at this moment, a liquid jet enters the chamber and the initial pressure (in the far field) is recovered. In fact, this liquid jet deforms the bubble (see 7 and 7’) and induces its violent collapse (see 8 and 8’), thus generating the emission of light pulses and mechanical vibrations as shown in Fig 6. The process between instants (7 and 8) and (7’ and 8’) is referred to as the second compression stage. It is important to mention that it was not possible to measure in detail the bubble collapse velocity during the second compression stage, since the videos show turbulent structures that do not allow to distinguish and follow the bubble surface. However, a crude and conservative estimate can be obtained by taking into account the time between instants (7 and 8) and the diameter of the chamber, thus the bubble collapse velocity ∼ 10 m/s. It is also important to note that in the final state of collapse, clouds and/or clusters of bubbles are observed, which could indicate that the bubble may possibly break-up before reaching a minimum volume. This issue will need to be clarified in future research. Fig 10 shows the plots of the bubble size evolution in the vertical direction (the corresponding axis is indicated in the first picture of the sequence for a bubble in the mixture, Fig 9) of each spheroid. It was observed that the vertical direction is predominant to describe the dynamics of compression and expansion of the cavities, for this reason and to facilitate the construction of the graphical results, the variation of the axes in the horizontal direction was omitted. All curves in Fig 10 were obtained using Tracker video analysis software [27]. Calibration was performed by taking the measurement of the cross-sectional diameter of the compression chamber (*ϕ*_*ch*_=3 mm) and the frequency rate at which the videos were taken (73,000 fps). The first thing to notice in Fig 10a is the gap between the two collapse times, which may be due to slight differences between the initial conditions of the bubbles as stated above. The second aspect to be clarified is the maximum size in the vertical direction reached by the bubble, which appears to be smaller than the diameter of the chamber. However, this is because the tip of the V-shaped end of the limb enters more towards the chamber reducing the maximum vertical height. Another interesting aspect is the asymmetry in the duration of the compression (from 1 to 4 and 1’ to 4’, respectively) and expansion (from 4 to 7 and 4’ to 7’, respectively) processes. For the bubble in water the compression time *t*_*c*_ is about 1.92 ms while the expansion time *t*_*e*_ is around 1.5 ms. In contrast, for the bubble in the mixture a compression time *t*_*c*_ ≈ 1.6 ms is observed while *t*_*e*_ ≈ 2 ms. Thus contraction (controlled by the closing power of the limb) is slower than expansion (due to “elastic” stored energy in the bubbles) in water and vice versa in the mixture. The reason for these differences is not clear and will be something worthy of further study. Fig 10b shows the graph of the bubble size speed in the vertical direction. The maximum velocities during the first compression stage are around—1 m/s, while for the expansion stage they are about 1.7 m/s. These values contrast with the 10 m/s estimated for the second compression stage. As expected, larger collapse velocities induce higher pressures and temperatures inside the bubble(s) along with photon production.

### Pressure developed in the compression chamber: Estimation via Rayleigh-Plesset equation

**Fig 11:**
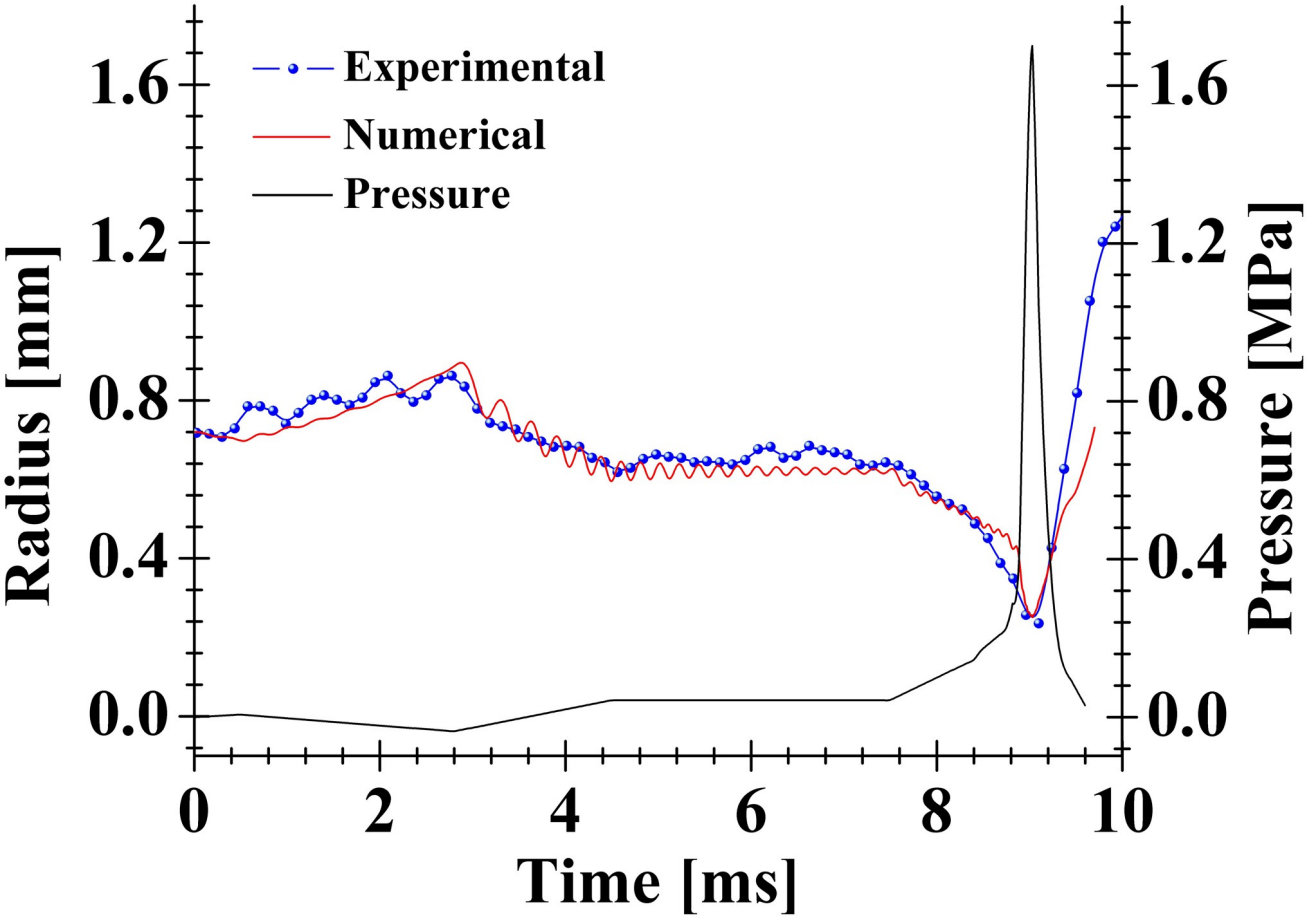
Comparison between theoretical and numerical curves of the bubble size evolution in the vertical direction. The experimental curve was obtained for the bubble seeded in water corresponding to the one in Fig 10a. The pressure curve obtained from the numerical model is also depicted.

Fig 11 shows a comparison between the experimental and numerical curves of the bubble size oscillations in the vertical direction (corresponding to Fig 10a). The numerical approximations were achieved via the Rayleigh-Plesset (R-P) equation (for spherical bubbles), which includes the liquid viscosity and the damping by acoustic radiation [28]:

$$\begin{array}{c} \begin{array}{c} \rho_{l}(R\ddot{R}+\frac{3}{2}{\dot{R}}^{2})=P_{1}-(P_{0}-P_{d}\left(t\right)), \end{array} \end{array}$$ (13)

subject to the initial conditions

$$\begin{array}{c} \begin{array}{c} R\left(0\right)=R_{0},\;\dot{R}\left(0\right)=0, \end{array} \end{array}$$ (14)

where the pressure in the liquid at the liquid-gas interface *P*_1_ is

$$\begin{array}{c} \begin{array}{c} P_{1}=P_{g}\left(R\right)+\frac{R}{3c_{g}}\frac{d}{dt}P_{g}\left(R\right)+P_{v}-\frac{2\sigma }{R}-\frac{4\nu \dot{R}}{R}, \end{array} \end{array}$$ (15)

with

$$\begin{array}{c} \begin{array}{c} P_{g}\left(R\right)=(P_{0}+\frac{2\sigma }{R_{0}}-P_{v}){\left(\frac{R_{0}}{R}\right)}^{3\kappa }, \end{array} \end{array}$$ (16)

and *R*, *P*_*g*_(*R*), *P*_*v*_, *σ*, *η*, *ρ*_*l*_, *P*_0_, *c*_*g*_ and *P*_*d*_(*t*)¸ are the bubble radius, gas pressure, vapor pressure, surface tension, liquid viscosity, liquid density, ambient pressure, the sound speed in the gas and driving pressure. An isothermal bubble was assumed, thus *κ* = 1. The value of the remaining parameters were set as: *ρ*_*l*_ = 1036 kg/m^3^, *η* = 0.001 Pa⋅s, *σ* = 0.073 N/m, *P*_*v*_ = 3000 Pa, *c*_*g*_ = 319 m/s, *R*_0_ = 0.72 × 10^−3^ m, *P*_0_ = 77, 000 Pa. Eq 13 is commonly used to describe the radial evolution of spherical bubbles knowing the driving pressure as a function of time [7]. Here we wish to estimate *P*_*d*_(*t*) from the experimental curve of the bubble radius. Of course, we are aware that the bubbles studied with the proposed device evolve in non-spherical shapes, therefore, the results presented in Fig 11 should be seen as a rough approximation. The function that describes *P*_*d*_(*t*) was constructed in such a way that the numerical curve of the radius *R*(*t*) approximated the experimental curve as closely as possible.

The procedure consists in considering multiple sub-functions to shape the *P*_*d*_(*t*) curve like a piecewise function. The first subfunction represents a straight line with a slight positive slope to simulate a small decrease in the initial bubble radius; this first segment starts at *t*=0 s and extends up to *t* = 0.4 ms. The slope of the first section was tuned until an acceptable agreement between the theoretical and experimental bubble radius curves was observed at [0 ≤ *t* ≤ 0.4 *ms*]. The second section has a negative slope to simulate an expansion of the bubble, this segment extends from *t* > 0.4 ms to *t* = 2.8 ms. The slope of the second segment was gradually varied until a good approximation between the numerical and experimental curves of the cavity radius was observed. It is important to note that the end point of the first segment was joined to the initial point of the second segment to achieve continuity. So, the *R*(*t*) curve sets the standard for establishing both the initial and final ends of each segment and the sign of its slope. In a similar way as described above, and in a progressive manner, the remaining segments were constructed until the *P*_*d*_(*t*) curve was formed. It is important to mention that this procedure was developed by trial and error without using a numerical fitting method; the achieved approximation was only judged by visual inspection.

As expected, the driving pressure depicts a pulse that would represent the pressure induced by the limb motion from its release to the closed position. It is interesting to note that the amplitude of *P*_*d*_(*t*) is about 1.7 MPa, this value is close to the lower limit of the pressures generated inside the channel-like cavity formed between the plunger and the socket in real snapping shrimps [29]. It should be emphasized, however, that the above estimate is only an approximation to guide us on the order of magnitude of the pressure generated by the mechanism. It will be necessary in future work to measure the pressure inside the chamber. One possibility would be to install PVDF film sensors [30] on one or both side faces of the compression chamber and to complement these measurements with simulations employing high-order schemes [31] to accurately describe the non-spherical collapse and the pressure field in the gas and liquid media (inside and outside the bubble).

As a final comment for this section, the bubble can grow large enough to adopt a cylindrical shape (imposed by the inner solid surface of the chamber, see snapshots 6 and 6’ in Fig 9) and at some instants it is likely to change from cylindrical to spherical as it collapses. For a crude description of the expansion/collapse dynamics in these cases one would have to implement the Rayleigh-Plesset equation for cylindrical bubbles [32] and even combine this approximation with the R-P equation for spherical cavities.

### Shape evolution of bubbles in water under different initial conditions

**Fig 12:**
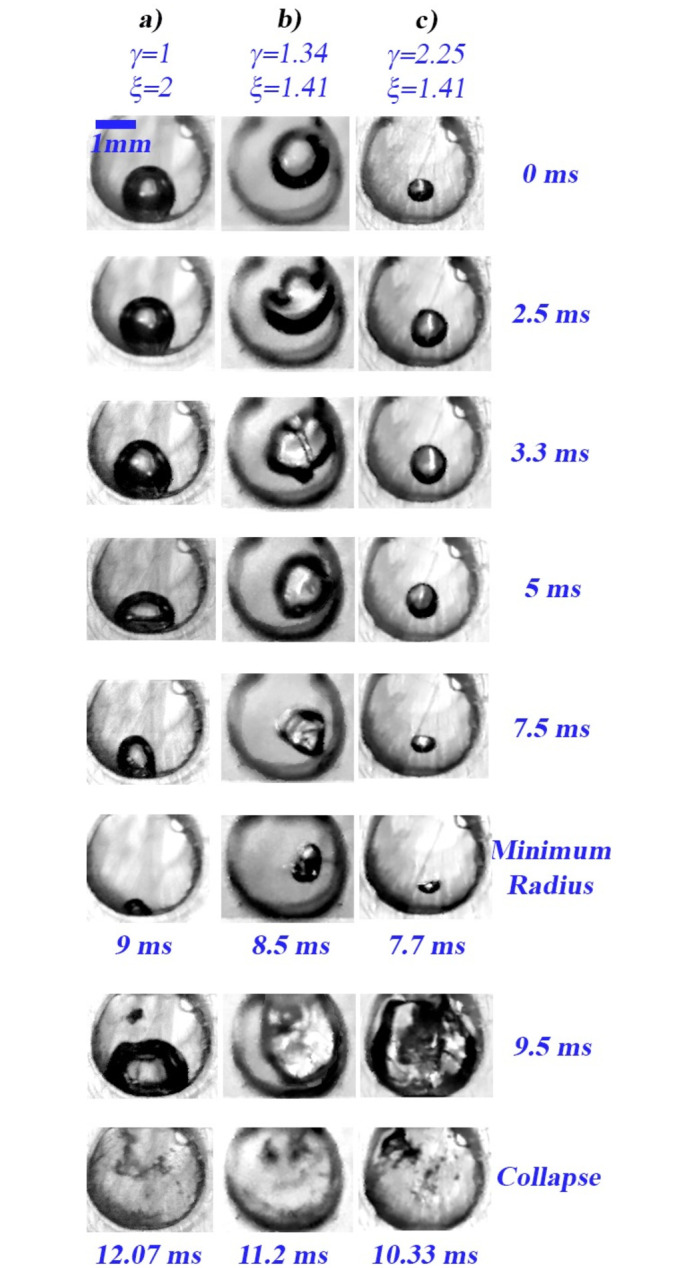
High-speed photographic sequences of the shape evolution of three bubbles in water under different initial conditions (different sizes and positions inside the chamber). Note that the bubble shape evolution depends on the standoff and curvature parameters. (a) Bubble oscillates volumetrically acquiring oblate/prolate spheroid shapes. (b) The formation of a microjet is clearly observed at 3.3 ms. (c) The bubble collapses into oblate/prolate spheroidal shapes until it reaches a minimum size with a typical flattened spheroid shape suggesting the formation of a microjet at 7.7 ms.

Fig 12 displays the shape evolution of three different bubbles. Fig 12a corresponds to the conditions *ϕ* ≈ 1.5 mm, *γ* = 1, *ξ* = 2; this bubble only contracts into non-spherical shapes without forming micro-jets. On the other hand, Fig 12b corresponds to the conditions *ϕ* ≈ 1.62 mm, *γ* = 1.34, *ξ* = 1.41; where both the generation of an air jet over the bubble (2.5 ms) and of a high-speed liquid jet (3.3 ms) during the first compression stage are observed. The air jet is an interesting phenomenon that might be due to initial non-sphericity, inertial effects and/or the interaction between the bubble gas-liquid interface and the solid boundaries [33–35]. The speed of the liquid jet was measured and gave a value of about 9.44 m/s implying Reynolds and Weber numbers of around *Re* = 1.6 × 10^4^, *We* = 2 × 10^3^, respectively. Note that these dimensionless numbers suggest an energetic jet with sufficient capacity to impact solid surfaces and cause erosion. It is worth noting that liquid jets with velocities in the same order of magnitude have been observed during the collapse of bubbles (driven by acoustic fields) near solid surfaces (0.9 < *γ* < 1.8) and also due to the implosion of cavities near free surfaces (*γ* = 0.56) [36–38]. Fig 12c corresponds to the conditions *ϕ* = 0.66 mm, *γ* = 2.25, *ξ* = 1.41; in this case the bubble experiences a contraction and at the same time a slight displacement towards the base of the chamber. A possible explanation for the latter is that the Bjerknes force weakens with increasing *γ*, resulting in a decrease in the distance between the bubble and the wall [39]. In all three cases an expansion stage is perceived after the bubbles have reached the minimum radius, then a second compression stage is observed together with the emission of photons, all of this occurs similarly to what is exhibited in Fig 6.

**Fig 13:**
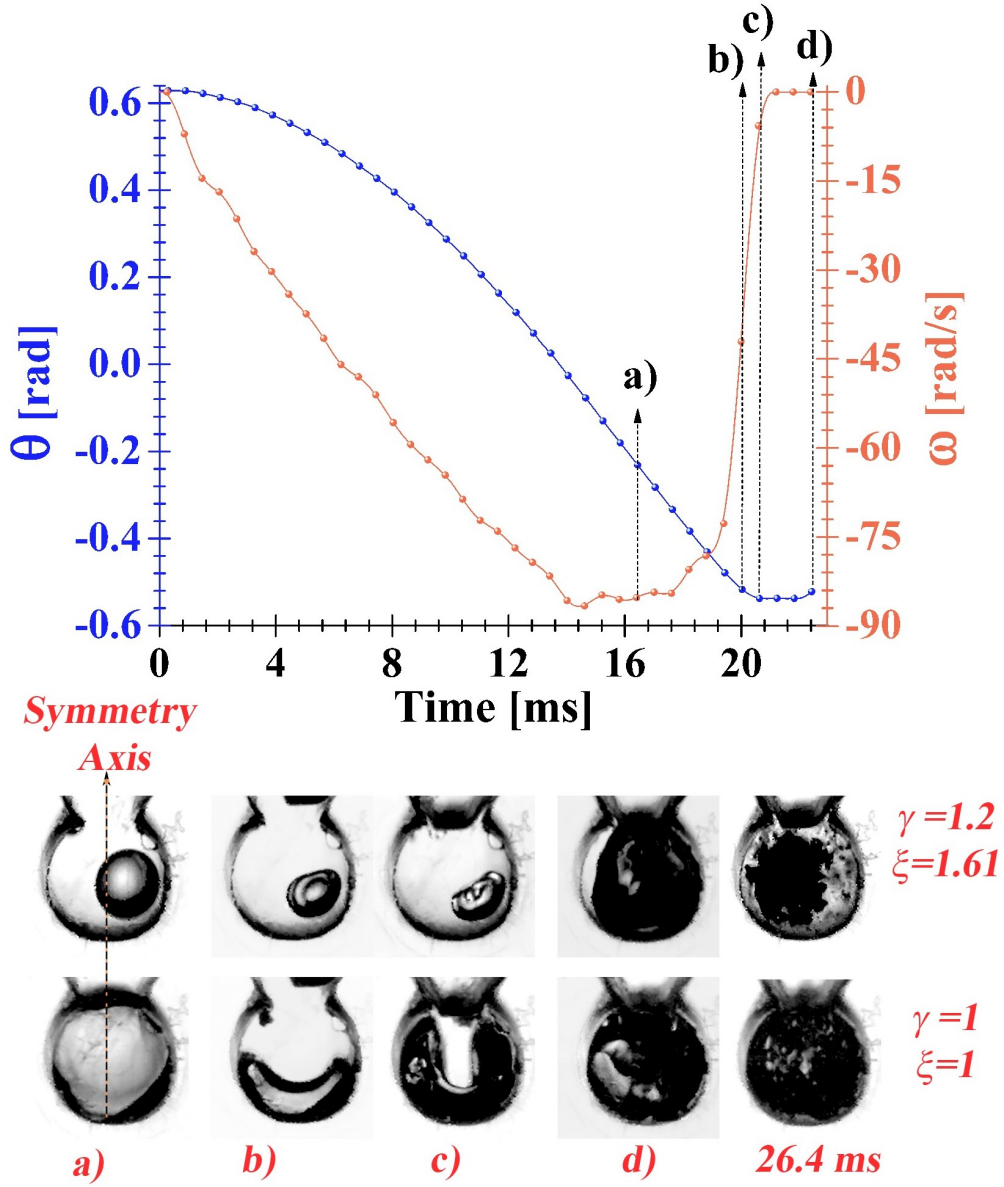
The upper part shows the curves of the displacement and angular velocity developed by the limb. In the lower part we can see the shape evolution of two bubbles at different initial conditions (distinct sizes and positions inside the chamber). The experiments were performed in water using two springs to generate the closing power.

Fig 13 shows the shape evolution of two air bubbles with different standoff and curvature parameters, both results are from tests performed in water with a closing power generated by two springs. From instant *t* = 0 to instant (a), both bubbles exhibit small-amplitude volumetric oscillations (like a beating) while maintaining approximately their original size, shape, and position. Afterward, from instant (a) to instant (b) the bubbles undergo a compression process, evidently the shape of the cavities evolves differently due to their different initial conditions. As expected, when the bubble is positioned off of the symmetry axis of the chamber, it will compress forming a liquid jet (this is also observed in the central sequence of Fig 12). From instant (b) to instant (c) the final stage of bubble compression occurs. The originally spherical cavity with *γ*=1.2 deforms developing a jet, just at instant (c), which appears to be of low intensity. On the other hand, the originally cylindrical bubble (since it occupies practically the whole chamber volume) with *γ*=1 deforms laterally giving rise to a flattened cylindrical bubble whose cross section looks like a horseshoe according to the accessible side view. In this case the asymmetric contraction seems to produce an incipient low energy jet. From instant (c) to instant (d) the bubbles expand until they reach the chamber walls, note that the bubbles develop irregular shapes. As pointed out before, it is observed the entrance of a liquid jet, at instant (d), causing the collapse of the irregular shape bubbles. However, in this case the implosion of the cavities does not generate luminescence and only the formation of bubble clusters exhibiting a series of expansions and collapses of low intensity is observed. It is important to note that the duration of the expansion (*t*_*e*_ ≈ 1.8 *ms*) and collapse (*t*_*c*_ ≈ 4.18 *ms*) processes give a ratio *t*_*c*_/*t*_*e*_=2.32, which indicates that the expansion phase is more than twice as fast as the collapse phase. It is also worth noting that the bubble contraction using two springs is less violent than that induced using four springs. In other words, by comparing the bubble collapse times in Fig 10a (in water 1.92 *ms* and in the mixture 1.6 *ms*) against those in Fig 12 (4.18 *ms*), it is clear that the power produced with four springs produces collapses about twice as fast as those developed with less closing power.

### Effect of initial radius and limb closing velocities on light emission

**Fig 14:**
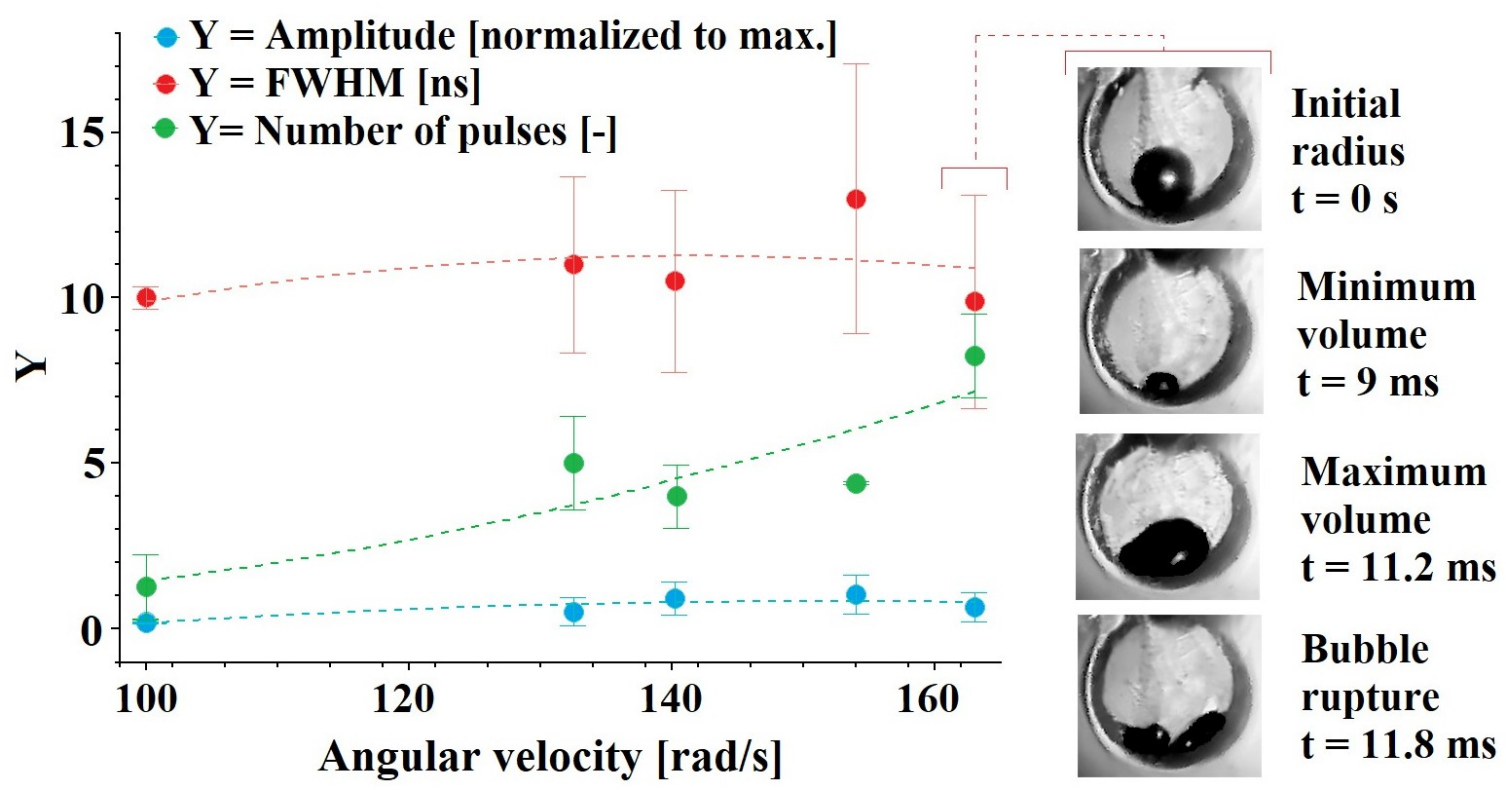
Average characteristics of the light pulses generated by the collapse of a bubble at different rates of limb closure. The photographic sequence on the right side shows the initial condition of the bubble at 0 s, as the arm is closing the bubble collapses to reach a minimum radius at 9 ms, then the cavity bounces and expands to reach a maximum volume at 11.2 ms displaying an irregular shape, finally the bubble collapses again and breaks into several bubbles at 11.8 ms. The error bar at each point represents the standard deviations from the mean for a set of three tests.

In this section, we report a study to establish the effect of some parameters on the characteristics of the light pulses coming from bubble collapses induced by the sudden closure of the limb. The parametric study contemplates two different types of experiments. In the first type, the initial bubble size was maintained in all trials and the closing speed of the limb was varied by using different spring arrangements (more springs and/or stiffer springs). The analysis of the results for this case focused first on identifying the light pulse with the highest amplitude for each closing event and then measuring its width. In this way, however, we observed a lot of variability without identifying clear trends. Instead, we focused on analyzing the widths and amplitudes of all light pulses generated by each closure event and in this way interesting trends were found. Fig 14 shows the number of pulses identified during each event and their corresponding average widths and amplitudes. As the closing velocity increases, the number of pulses increases, however, the widths and amplitudes seem to reach a maximum (at ≈ 145 rad/s) and then decrease slightly. This behavior can be explained by analyzing the videos of the bubble and limb dynamics. What is observed is that as the closing speed increases, the tip of the V-end of the limb stops bouncing (due to the force exerted by the springs that remain pre-tensioned when the limb reaches the closed position) sealing the compression chamber completely; when this regime is reached, the bubble shows only a first collapse followed by a rebound until it reaches maximum expansion and then collapses a second time and breaks up asymmetrically. The rupture process results in the formation of unequally sized daughter bubbles (in most cases, two were observed, but in other cases more than two cavities were observed), whose collapse no longer appears to be as violent as that observed in bubbles that do not break when their collapse is induced by closing velocities less than 140 rad/s. Despite this, it is likely that the contraction of bubbles produced by rupture is sufficiently intense to generate photon emission, thus, each bubble represents at least one emission point explaining the increase in the number of pulses with increasing arm closing velocity. The study of bubble breakup in the final stages of collapse produced with the present device may be of great interest to extend the understanding of rupture mechanisms such as Rayleigh-Taylor instabilities [40, 41].

**Fig 15:**
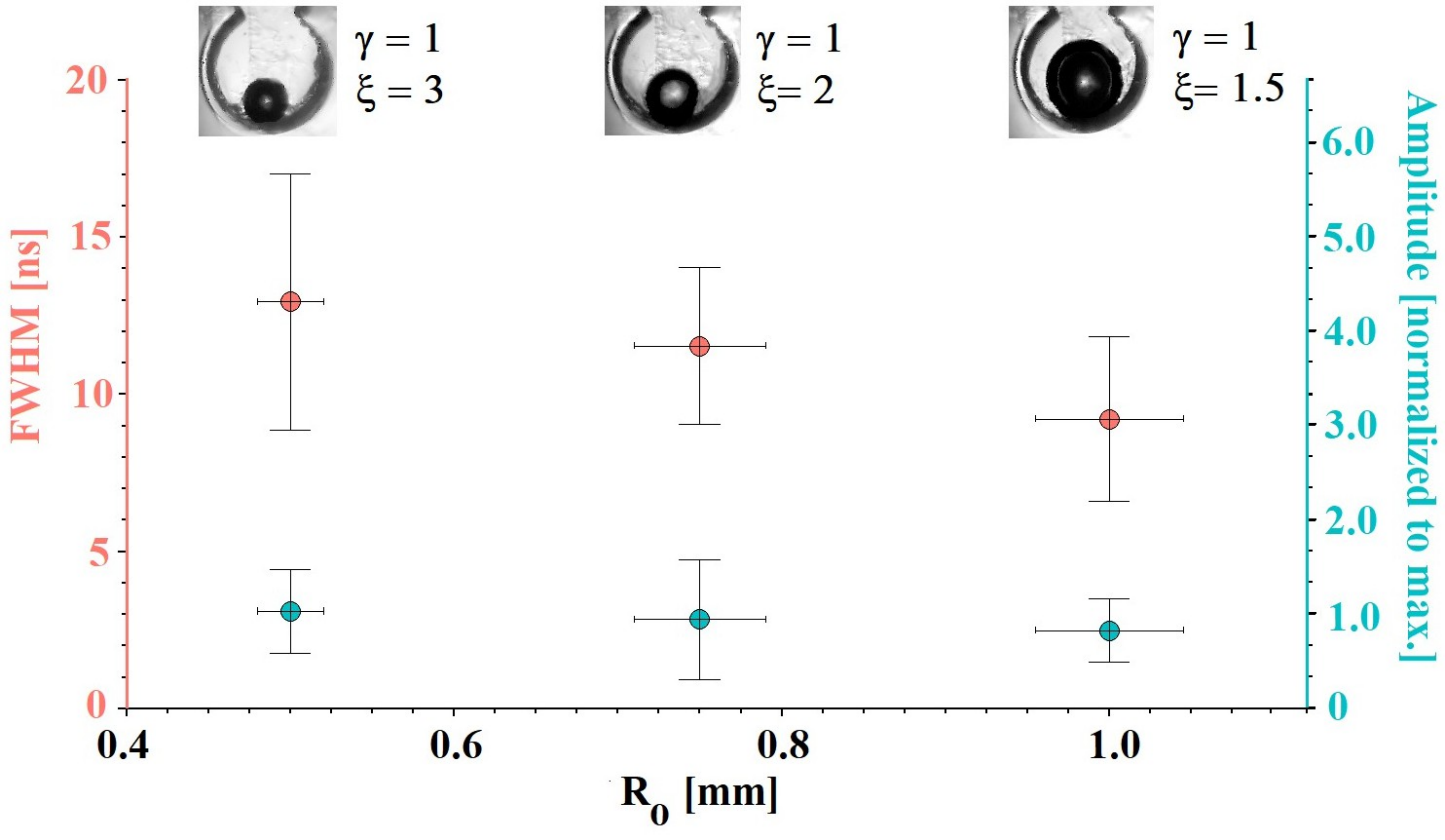
Average characteristics of the light pulses obtained with the same speed of the limb considering three initial bubble sizes. The error bar at each point represents the standard deviations from the mean for a set of three tests.

In the second type of experiments, the initial size of the bubble was varied by controlling the amount of gas inside the bubble and maintaining the same closing speed (140 rad/s). Fig 15 shows the results corresponding to this second type of tests. Three different bubble sizes were considered (*ξ* = 1.5, 2, 3) and for easiness all cavities were positioned at the bottom of the compression chamber to maintain a fixed initial position. Our attention was focused on the analysis of all the pulses emitted by each event and what was obtained and plotted are the average amplitudes and widths. As the initial bubble size increases, the average pulse amplitude decreases. This was to be expected, since by keeping the closing power fixed, larger bubbles tend to collapse less violently and therefore emit pulses of smaller amplitude. On the other hand, the average width of the light pulses remains almost constant.

## Conclusions

A mechanical device equipped with a fast-closing rotational limb was built and tested. The proposed design was inspired by the sudden motion of both the pistol shrimp claw and the mantis shrimp appendages. The proposed mechanism also incorporates key features from the U-tube device, but instead of using a liquid piston, one end of the limb has a V-shaped geometry serving as a plunger that fits into a V-shaped socket whose tip connects to a rigid wall chamber. An air bubble is seeded in the chamber and when the limb is released a pressure field (first with small fluctuations around the ambient pressure and then the formation of a pulse) is generated inducing changes in the dynamics of the bubble shape evolution. By changing the standoff and curvature parameters, as well as the closing power of the limb it is possible to control the dynamical behavior of the cavity. In a first set of experiments, four stages describing the dynamic behavior of the bubble were found:

1. A slight expansion-contraction stage accompanied by very weak volumetric oscillations.
2. First compression stage. The formation of gas and liquid micro-jets is observed when the vertical symmetry axis of the bubble is initially located outside of the chamber symmetry axis, on the other hand, when there is a coincidence between these axes, the bubble only contracts exhibiting non-spherical shapes, alternating between oblate and prolate spheroidal structures.
3. An expansion stage after the bubble reaches its minimum radius. At the end of this stage the bubble surface reaches the walls of the chamber and clearly presents irregular shapes on its surface. In most of the cases analyzed, the expansion stage is faster than the first compression stage.
4. Second compression stage. This process begins when the limb rebounds and stops sealing the chamber allowing a jet of liquid to enter from the surrounding medium, inducing a violent collapse. Just when the bubble reaches the minimum volume a flash of light is produced. The light pulses were monitored in water and in a mixture of water and chlorine dioxide; a small addition of this substance enhances the light intensity and produces shorter pulses.

In a second set of experiments, the effect of initial bubble radius and limb closing velocities on the characteristics of the light emission was explored. As the closing speed increases while keeping the bubble size constant, the number of light pulses increases; however, the widths and amplitudes appear to reach a maximum and then decrease slightly. Such an increase in the number of light flashes may be due to the rupture of the originally seeded bubble, which results in the formation of secondary bubbles. The collapse of these may be intense enough to generate light from different emission points, which would explain the increase in the pulse count. On the other hand, as the initial bubble size increases while the limb-closing power is held constant, a decrease in the mean amplitude of the light pulses is observed. This is to be expected, as larger bubbles tend to collapse less violently and therefore emit lower amplitude pulses.

The proposed device is a novel alternative to produce and study cavitation and its effects near and on solid surfaces (e.g. erosion and/or corrosion), as well as phenomena such jets and luminescence, among others. The apparatus presented here could be useful for further study of the dynamics of liquid jets (such as those observed in Figs 9, 12 and 13), and from this one could better understand their erosive effects on different materials [42] or to develop applications oriented to drug deposition in tissues [43]. Other possible applications, which would be interesting to explore and refine with the device, are those that take advantage of UV light emission from the collapse of air bubbles in water to inactivate pathogens [44]. Additional attractive features of the apparatus are its low manufacturing cost, simple design, and compact size for experimental studies.

## Supporting information

S1 Video Dynamics of limb closing. (AVI)

S2 Video Shape evolution of a bubble in water. The bubble oscillates volumetrically acquiring oblate/prolate spheroidal shapes (this corresponds to the sequence of photos in column (a) in Fig 12).
(AVI)

S3 Video Shape evolution of an air bubble in water. At the beginning of the test, the bubble is placed outside the axis of symmetry of the chamber, and thus the bubble is compressed forming a jet (this corresponds to the sequence of photos in the top row of Fig 13).
(AVI)
